# Burosumab an emerging therapy in TIO: a local clinical survey and a systematic review with individual patient data analysis

**DOI:** 10.3389/fendo.2026.1804013

**Published:** 2026-04-02

**Authors:** Anita Vergatti, Veronica Abate, Aquilino F. Zarrella, Natalia Fieramosca, Gianpaolo De Filippo, Daniela Merlotti, Luigi Gennari, Ciro Menale, Lanfranco D’Elia, Domenico Rendina

**Affiliations:** 1 Department of Clinical Medicine and Surgery, Federico II University, Naples, Italy; 2 Service de médecine interne et rhumatologie, Service de Santé des Armées, Hôpital National d’Instruction des Armées Percy, Clamart, France; 3 Department of Medical Sciences, Azienda Ospedaliera Universitaria Senese, Siena, Italy; 4 Department of Medicine, Surgery and Neurosciences, University of Siena, Siena, Italy

**Keywords:** burosumab, hypophosphatemia, phosphaturic mesenchymal tumor, systematic review, tumor induced osteomalacia

## Abstract

**Introduction:**

Burosumab, a recombinant antibody against fibroblast growth factor 23 (FGF23), is a new therapeutical option for Tumor induced osteomalacia (TIO). To estimate the clinical need and to evaluate the efficacy and safety of burosumab, we conducted a hybrid study composed by a clinical survey and a systematic review.

**Methods:**

All patients referring for TIO to the Federico II University were enrolled in our clinical survey. A comprehensive literature search on a Medline, Google Scholar, Google Books, and the Cochrane Library was conducted for the Systematic Review. Data were extracted on 12/31/2025.

**Results:**

For the local survey, we collected 10 patients affected by TIO [M: F = 5 (50.0%): 5 (50.0%); mean age at symptom onset 57.6 ± 14.3 years]. Two (20.0%) patients received burosumab, obtaining clinical and biochemical improvement. Two who didn’t receive the treatment died. For the systematic review, we collected 42 studies and 49 cases [M: F = 19 (44.2%): 24 (55.8%); mean age at onset 36.8 ± 21.3 years; mean age at diagnosis 44.5 ± 22.9 years]. Burosumab reduced clinical symptoms and ameliorates biochemical investigations by normalizing phosphate serum levels. It is also safe and well tolerated.

**Conclusion:**

Burosumab represents a safe and efficient treatment in TIO patients, to be administered in case of tumor identification while waiting for surgery, and in case of surgical inoperability to manage hormonal FGF23 effect.

## Introduction

Tumor induced osteomalacia (TIO) is a rare paraneoplastic syndrome, caused by over-production of fibroblast growth factor 23 (FGF23) from a phosphaturic mesenchymal tumor (PMT). FGF23 is 32 kDa phosphaturic hormone produced in bone (1). The FGF23 excess leads to phosphate renal depletion and vitamin D reduced activation, finally resulting in osteomalacic syndrome (2–4). This condition is clinically manifested by bone pain, fractures and fatigue, that impairs life quality (5), other than symptoms related to the presence of the tumor.

PMTs are usually benign and small tumors, tricky to identify with a common radiological approach and mostly requiring a systemic venous sampling for FGF23 or a composite radiological image, as ^68^Ga-DOTATOC Positron Emission Tomography (^68^Ga-DOTATOC PET/CT) or ^99m^Tc HYNIC-TOC single-photon emission CT-CT (2). In case of a full diagnosis of the causal tumor, first line treatment is represented by surgical removal in case of resectability (6). Otherwise, a life-long medical support with phosphate and calcitriol was and still remains the only option for such a case (7). Recently, the innovation in the pharmaceutical field resulted in the engineering of burosumab (International Nonproprietary Name: burosumab; code/synonyms: KRN23), a recombinant fully human monoclonal IgG1κ antibody against FGF23 (8). Burosumab inhibits the excess of circulating FGF23, inducing an improvement of clinical and biochemical parameters (7). The introduction of burosumab changed the course of patients affected by FGF23 excess, by replacing a several times a day oral treatment with a once every two- or four-weeks subcutaneous injections (9). In children affected by X-linked rickets, genetically determined and characterized by FGF23 over-production (10), burosumab was proved to improve rickets, growth, lower limb deformity, and mobility. On the contrary, due to the rarity of condition, such improvement is currently just sporadically reported in patients with TIO, but systematically described just in patients with X-linked rickets (11). At the time, burosumab has been evaluated in two studies enrolling a total of 27 patients with TIO (12, 13).

The present study aims to address the gap in post-marketing clinical evidence through a hybrid design study, composed by a case report, a clinical survey from our center experience and a systematic review including all the reported cases of TIO patients treated with burosumab (14–16).

## Methods

### Clinical survey

From January 1^st^, 1998 to December 31^st^, 2025, all patients referred to the Department of Clinical Medicine and Surgery of University of Naples for TIO were enrolled in our clinical survey. Five of the patients included in this survey had already been previously reported in two earlier publications (17, 18). All patients diagnosed with TIO underwent a comprehensive evaluation of phosphate metabolism and were subjected to systemic venous sampling for FGF23 and an imaging examination to localize the tumor producing FGF23 or sites of increased tracer uptake suggestive of PMTs, respectively. A standardized pre-piloted form was used to extract relevant data from enrolled patients’ medical records. The extracted data included: sex, symptoms, age at symptom onset, age at the diagnosis of TIO, serum phosphate levels, serum calcium levels, parathormone (PTH) levels, intact FGF23 levels and/or c-term FGF23 levels, tubular maximum reabsorption of phosphate to glomerular filtration rate ratio (TmP/GFR), Tubular reabsorption of phosphate (TrP), alkaline phosphatase levels (ALP), 25hydroxyvitamin D (25OHD) levels, conventional therapy and duration of conventional therapy, TIO localization, operability. Two authors (AV and VA) extracted data independently, and any discrepancies were resolved through discussion with author (DR). Written informed consent for clinical data collection and anonymized analysis was obtained from all patients, in accordance with institutional regulations and the principles of the Declaration of Helsinki.

### Systematic review with individual patient data analysis

This systematic review was registered in PROSPERO (registration number: CRD420251275425).

#### Data sources and search strategy

**Figure 1:**
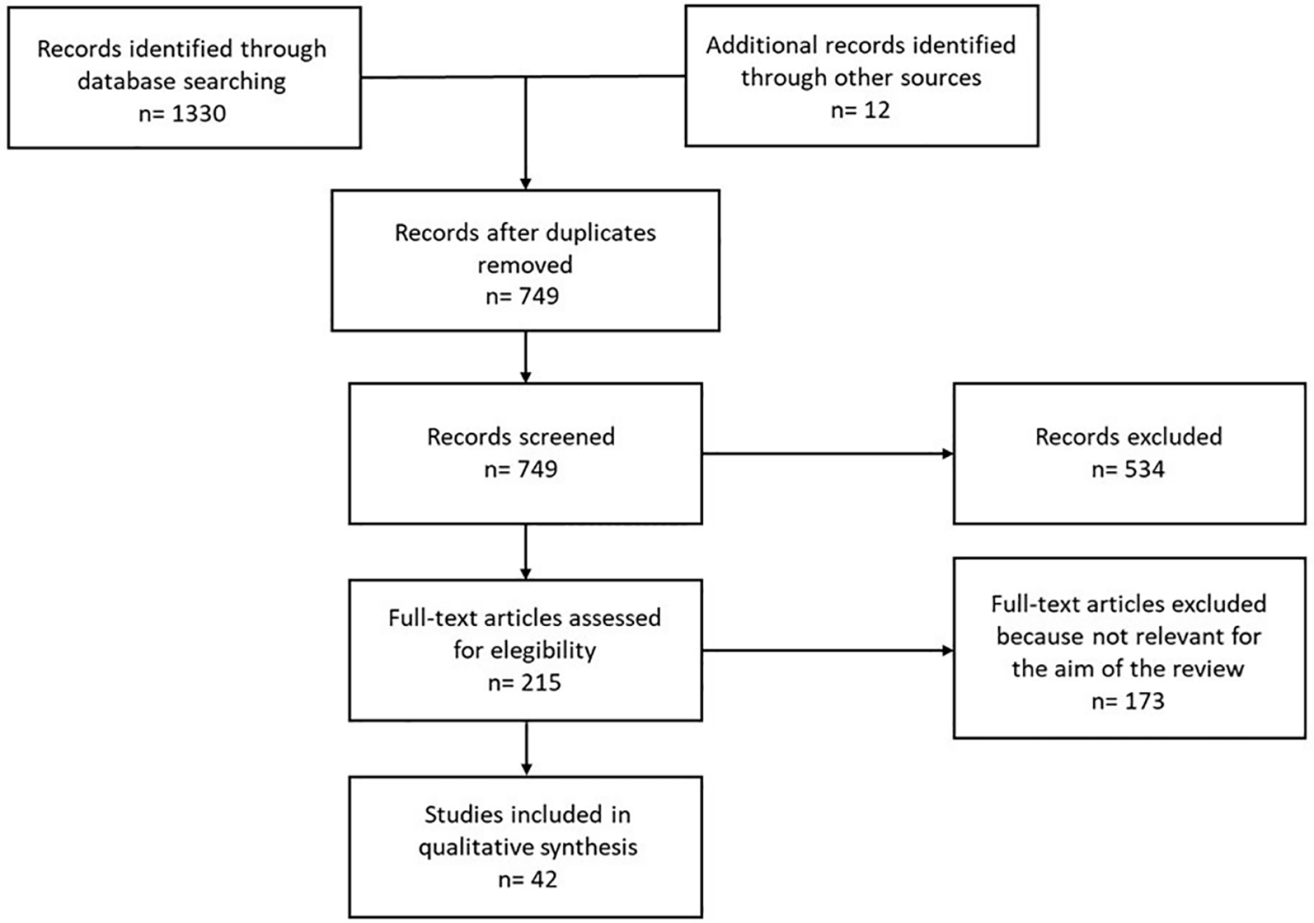
Study flow chart.

This systematic review was designed in accordance with the Preferred Reporting Items for Systematic Reviews and Meta-Analyses (PRISMA) 2020 guidelines (**Figure 1**) (19). A comprehensive literature search was conducted across Medline, Google Scholar, and the Cochrane Library on December 31^st^, 2025. Google Books was also took into account, to search for additional cases into references. The search strategy included the terms “tumor-induced osteomalacia,” or “oncogenic osteomalacia,” or “hypophosphatemia,” or “burosumab.” No restrictions regarding language were applied. In addition, the reference lists of all eligible articles were manually screened to identify further relevant studies.

#### Study selection

Eligible studies comprised case reports, case series and clinical trials. All selected articles were retrieved in full text, and their reference lists were carefully examined to identify and exclude duplicate data. Predefined inclusion criteria encompassed patients of any age with a clinical diagnosis of TIO. Exclusion criteria included patients of any age diagnosed with primary hyperparathyroidism, Fanconi syndrome, or genetic disorders affecting phosphate homeostasis (20).

#### Data extraction

From December 31^st^, 2025 to January 15^th^, 2026 studies were analyzed and data extracted, as follows. Titles and abstracts, when available, of all records identified through the predefined search strategy were independently screened by two reviewers (AV and AFZ) to determine potential eligibility based on the predefined inclusion criteria. The full texts of potentially relevant studies were subsequently retrieved and independently evaluated for eligibility by two reviewers (AV and AFZ). Studies published in languages other than English, French, or Italian (including Chinese, Russian, Spanish, Portuguese, German, Hungarian, and Japanese) were translated into English or Italian by a professional translator. Disagreements regarding study eligibility were resolved through consensus among all members of the review team. Data extraction was performed using a standardized data collection form. Extracted variables included: first author name, publication year, sex, age at symptom onset, age at diagnosis of TIO, age at burosumab treatment initiation, pre- and post-burosumab serum phosphate levels, pre- and post-burosumab serum calcium levels, pre- and post-burosumab PTH levels, pre- and post-burosumab intact FGF23 levels and c-term FGF23 levels, TmP/GFR, TrP, pre- and post-burosumab ALP, pre-burosumab 25OHD levels, pre- and post-burosumab 1,25 dihydroxy vitamin D [1,25(OH)2D] levels, burosumab dosage, follow-up, pre-burosumab conventional therapy, duration of pre-burosumab conventional treatment, adverse drug reaction (ADR) (21), burosumab treatment discontinuation, clinical symptom resolution, TIO localization, imaging for tumor localization, and operability. Data were independently extracted by two reviewers (AV and VA), with discrepancies resolved through discussion involving DR and GDF. When necessary, missing information was requested directly from the study authors. Methodological quality assessment of the included case reports and case series was conducted using the Joanna Briggs Institute critical appraisal checklists for case series (**Supplementary Materials: Table 1**) and case reports (**Supplementary Materials: Table 2**) (22). To assess the methodological quality and risk of bias of the randomized controlled trials included in this review, we used the Risk of Bias in Non-randomized Studies of Interventions (ROBINS-I) tool (23).

### Adverse drug reactions definition

ADR is an adverse event with a causal relationship to a drug. ADRs can be classified into different grades according to clinical severity. Mild ADR (Grade 1) doesn’t require any intervention; moderate ADR (Grade 2) requires medical intervention or a change in therapy; severe ADR (Grade 3) results in hospitalization; life-threatening ADR (Grade 4) requires urgent intervention; and death (Grade 5) (24).

### Statistical analysis

Statistical analyses were performed using IBM SPSS Statistics, version 28 (IBM Corp., Armonk, NY, USA). Data extracted from the included publications were entered into a single database and reanalyzed. Continuous variables were summarized as mean ± standard deviation (SD) or median with interquartile range [IQR; 25th -75th], as appropriate, while categorical variables were reported as absolute numbers and percentages. Due to the non-normal distribution of continuous variables and the limited number of paired observations, comparisons between pre- and post-burosumab biochemical parameters were performed using the Wilcoxon signed-rank test. Missing data were not imputed, and analyses were conducted on available cases only. All statistical tests were two-sided, and a p value < 0.05 was considered statistically significant. Given the descriptive nature of the study and the heterogeneity of the included case reports and case series, no multivariable or regression analyses were performed.

## Results

### Clinical survey

We collected data from 10 patients affected by TIO [M: F = 5 (50.0%): 5 (50.0%); mean age at symptom onset 57.6 ± 14.3 years]. All patients reported pain and functional impairment, and fractures were present in 7 (87.5%) patients. All patients exhibited hypophosphatemia (mean 0.43 ± 0.2 mmol/L), while serum calcium levels were within the normal range (mean 9.1 ± 0.5 mg/dl), and intact FGF23 levels exceeded 100 pg/mL. Mean ALP levels were 349.3 ± 187.6 U/L, mean PTH levels were 256.8 ± 47.9 pmol/L, and mean 25OHD levels were 90.4 ± 18.2 nmol/L (all patients were treated with Cholecalciferol according to Holick (25).

Six underwent ^68^Ga-DOTATOC PET/CT, 2 underwent Fluorine-18 fluorodeoxyglucose positron emission tomography/computed tomography (¹^8^F-FDG PET/CT), and 2 underwent octreotide scan. All patients underwent systemic venous sampling for FGF23 screening of concentration gradient, either to confirm or make a diagnosis.

Tumor localization was achieved in 7 patients, and the main sites involved were: vertebra, femur, tibia, lung, and brain. Tumor localization was not achieved in 3 patients, despite regular imaging follow-up.

Six patients had been treated with oral phosphate salts and calcitriol, with a mean follow-up of 5.7 ± 3.8 years.

Surgical treatment was performed in 4 patients (out of 7 with tumor identified), with a rapid and complete resolution of clinical symptoms and normalization of serum phosphate levels. Two patients died because of unrelated TIO causes, before burosumab became available for the treatment of TIO: one due to a haemorrhagic stroke and one due to occurrence of pulmonary sarcoma. Three patients received a prescription for burosumab. The tumor was identified in all of them (3 out of 7). Patients received a surgical consultation, that judged in all of them the tumor as inoperable. In one case the tumor was located to the tibia, and in the other two the tumors were multifocal and both located in the vertebras. Of patients receiving the burosumab prescription, two patients are currently receiving treatment with burosumab (starting at a dose of 0.3 mg/kg every 4 weeks). One is about to initiate burosumab. The last patient is still undergoing conventional therapy.

For the patients treated with burosumab, in accordance with the prescription criteria, follow-up examinations were performed after one month. All patients showed an improvement in phosphate levels (Δ-phosphate +0.67 ± 0.3 mmol/L), osteomalacia symptoms, with no ADRs.

Due to the limited number of patients treated with burosumab in our cohort, a formal comparative analysis between surgical and pharmacological treatment was not feasible.

### Systematic review with IPD

As showed in **Figure 1**, 42 studies were included in qualitative and quantitative syntheses. The case reports and the case series included in the final analysis were 37 and 4, respectively. Moreover, a clinical trial was included in the analysis. A complete list of studies included and their critical appraisals are reported in **Supplementary Materials** (**Supplementary Tables 1**, **2**). A total of 49 cases were included in the analysis. The mean age at symptom onset was 36.8 ± 21.3 years. Mean age at diagnosis of TIO was 44.5 ± 22.9 years, while mean age at initiation of burosumab therapy was 45.8 ± 21.6 years. Sex was reported in 43 cases, including 19 (44.2%) males and 24 (55.8%) females. The 83.4% of patients developed TIO after completion of growth, whereas 8 cases occurred during the growth phase.

### Clinical presentation

Clinical symptoms were reported in 44 cases. Thirty-two patients presented with osteomalacia syndrome (26), 8 with pain associated with functional impairment, and 3 with bone pain alone.

### Biochemical findings

All biochemical parameters pre- and post-burosumab are reported in **Table 1**. At diagnosis, all patients presented with severe hypophosphatemia (27) at diagnosis (**Table 1**). After burosumab treatment, normalization of serum phosphate levels was achieved in all but two patients. The mean increase in serum phosphate (Δ-phosphate) was +0.73 ± 0.29 mmol/L (95% CI: 0.61–0.86; p < 0.001). The Wilcoxon signed-rank test confirmed a significant improvement (p < 0.001) with a large effect size (r = 0.88). Median serum phosphate increased from 0.48 mmol/L [0.39–0.60] before treatment to 1.22 mmol/L [1.03–1.42] after burosumab (n=18; p < 0.001). A stratified analysis according to study design was also performed. Among patients derived from case reports, series and clinical trial serum phosphate significantly increased after burosumab therapy. Overall, the direction of biochemical improvement was consistent across study designs.

**Table 1:** Biochemical characteristics of the study populations.

Variables	Pre-burosumab	Post-burosumab	Available cases (n)	Δ	95% CI	P-value	Effect size	Paired cases (n)
Phosphate (mmol/L)	0.5 ± 0.2	1.3 ± 0.3	Pre: 41 Post: 19	+0.73	0.6-0.9	<0.001	0.88	18
TmP/GFR (mmol/L)	0.5 ± 0.2	–	Pre: 24	–	–	–	–	–
TrP (%)	67.2 ± 21.3	–	Pre: 10	–	–	–	–	–
Calcium (mg/dL)	9.3 ± 0.5	9.5 ± 0.4	Pre: 25 Post: 6	-1.3	-0.4-+0.2	0.45	0.39	6
25OHD (nmol/L)	74.0 ± 43.0	–	Pre: 25	–	–	–	–	–
1,25(OH)_2_D (pmol/L)	77.1 [59.2- 111.2]	150.9 [79.7- 268.9]	Pre: 22 Post: 8	+154.9	-201.0-+510.8	0.32	0.56	8
PTH (pmol/L)	273.3 ± 266.7	163.9 ± 88.2	Pre: 23 Post: 6	-19.8	-144.6-+104.9	0.75	0.32	6
Int FGF23 (pg/mL)	196.0 [108.3- 361.0]	–	Pre: 29 Post: 1	–	–	–	–	–
c-term FGF23 (RU/mL)	307.9 ± 176.2	–	Pre: 4	–	–	–	–	–
ALP (U/L)	519.6 ± 388.2	201.8 v 111.5	Pre: 24 Post: 11	-342.8	-449.9- -235.7	<0.05	0.91	11

Data are reported as mean ± SD or median (interquartile range), as appropriate. Statistical comparisons were performed using the Wilcoxon signed-rank test including only paired observations. Analyses were performed on available cases only due to missing data. TmP/eGFR: tubular maximum reabsorption of phosphate to glomerular filtration rate ratio; TrP, tubular reabsorption of phosphate; 25OHD, 25 hydroxy vitamin D; 1,25 (OH)_2_D, 1,25 dihydroxy vitamin d; PTH, parathormone; Int FGF23, intact fibroblast growth factor 23; c-term FGF23, c terminal fibroblast growth factor 23; ALP, alkaline phosphatase.

Moreover, a sensitivity analysis was performed excluding the patient derived from clinical trial and those derived from studies considered at high risk of bias (**Supplementary Tables 1**, **2**). Among the remaining patients (n = 17 with paired phosphate data), serum phosphate significantly increased after burosumab therapy (mean increase +0.72 mmol/L, p < 0.001), with a large effect size (r = 0.87).

### Pre-burosumab treatment

Data on pre-burosumab therapy were available for 35 patients. Two patients received no treatment, 30 were treated with calcitriol plus phosphate supplements, and three received phosphate supplements alone. The median duration of conventional therapy prior to burosumab initiation was 42.0 [15.0– 81.0] months.

### Tumor localization and operability

Tumor localization was reported in 26 cases and included lesions in the skin (6), femur (4), brain (3), ribs (3), vertebrae (2), sacrum (2), and, less frequently, the humerus (1), mastoid (1), skull (1), scapula (1), foot (1), and pelvis (1). Imaging used for tumor identification were reported in 34 cases and included ^68^Ga-DOTATOC PET/CT (22), ¹^8^F-FDG PET/CT (6), magnetic resonance imaging (3), and octreotide scintigraphy (3). Operability was reported in 39 cases: tumour was considered operable in 14 (35.9%) patients and non-operable in 25 (64.1%). Among the 38 cases with additional clinical information available, the most frequent outcome was failure to localize the tumor (11), followed by non-resectable localization (8), recurrence (5), and complete remission after surgery (5). Less commonly, patients experienced lack of post-surgical resolution (3), Cutaneous Skeletal Hypophosphatemia Syndrome (3), patient refusal (2), or had surgery still pending at the time of evaluation (1).

### Burosumab dosage and clinical outcomes

Burosumab dosage data were available for 28 cases with a mean dose of 0.7 ± 0.4 mg/kg. The mean follow-up duration was 23.3 ± 17.7 months and was reported in 23 cases. Post-treatment clinical outcomes were described in 41 cases, and a complete resolution of TIO-related symptoms was observed in all reported patients.

### Safety and treatment discontinuation

Information on ADRs was available for 31 patients. No adverse events were reported in 27 (87.1%) cases, while four patients experienced ADRs, but none were classified as Grade 3, 4 or 5. Treatment discontinuation data were available for 32 patients. Burosumab was discontinued in 5 (15.6%) cases, all of whom subsequently underwent surgical treatment, while 27 (84.4%) patients continued therapy without surgical removal.

## Discussion

To our knowledge, this is the first hybrid study assessing the clinical evolution of TIO patients receiving burosumab, by using a local clinical survey and a systematic review an individual patient data analysis. According to our clinical survey, about 30% of patients were eligible for treatment with burosumab. Patients who didn’t receive nor burosumab nor surgery, but only conventional therapy with phosphate and calcitriol had a higher mortality rate. On the other hand, the systematic review showed that treatment with burosumab reduces clinical symptoms and ameliorates biochemical investigations by normalizing phosphate serum levels. It is also safe and well tolerated.

A similar approach was attempted with patients affected by X-linked rickets. Indeed, a meta-analysis identified both the beneficial and the side effects of long standing burosumab treatment (11). Compared to X-linked rickets, patients with TIO had a similar beneficial effect in term of serum phosphate, and similarly tolerated the treatment, both because of the lack of site reaction events and of systemic adverse effects. In both conditions, burosumab allowed conventional treatment discontinuation, reducing the risk of common side effects. Indeed, long lasting treatment with calcitriol and phosphate salts lead to nephrocalcinosis and nephrolithiasis (28) and tertiary hyperparathyroidism (29), furtherly complicating the clinical management. In addition, the similar efficiency was obtained with a four weeks administration in TIO patients and with a two weeks administration in X-linked rickets for burosumab, and with a three times per day oral administration with conventional therapy.

Furthermore, contrary to X-linked rickets, burosumab presents multiple advantages in TIO patients.

Despite a complete surgical removal of PMTs is always recommended whenever possible, burosumab works by reducing symptoms, as demonstrated in the current study. However, in some cases as for unresectable tumors, multifocality, recurrence or metastasis, burosumab represent a tailored therapy, aiming to both ameliorate life quality, efficiently correct the hypophosphatemia and reducing the side effects of conventional therapies. In addition, in a previous study, we described the malignant potential of PTMs, mostly in case of high FGF23 serum levels (3). The inclusion of burosumab as tool to bind FGF23 and possibly reduce its potential malignant effect, foreshadow a hopeful reduction of tumor evolution for which further studies are needed to confirm.

It is also been suggested in 5 cases (30–33) treatment with burosumab while waiting for surgery, obtaining a complete resection and most importantly healing of the patients without any complications. This approach may be applied to all the TIO patients waiting for surgery to reduce FGF23 systemic effect and hopefully surgical complications. However, to prove this point, more studies are needed.

One further remark must be underlined. In our clinical survey 3 patients out of 10 (30%) were eligible for burosumab, but only two were under it at the time, obtaining clinical and biochemical improvement in these lasts. For this reason, our clinical survey provided a valuable real-world insight of burosumab prescription in a third level center according to European Medicine Agency (EMA) indication. Indeed, patients are eligible to treatment in case of unresectable tumors or of unlocalizable tumors in adults and in children (34). In return, this markedly limits inferential strength regarding clinical efficacy. On the contrary in a recent study, authors identified a population of 73 subjects out of 1566 (4.7%) who couldn’t go through surgical intervention because the tumor remained un-identified, and a population of 57 out of 1070 (5.5%) because surgery was considered impossible or not resolutive (2). According to above, burosumab should have been prescribed in all of them, but just 28 subjects received the drug in favor of alternative therapeutic approaches without any successful outcome. So that, more efforts are required to improve burosumab indications and dissemination to furtherly ameliorate TIO patient clinical history.

As last, results may have took advantage of the data from patients who didn’t perform surgery despite tumor identification (i.e. refusal, inoperability), that are not available in our clinical survey yet, and lack in systematic review. Follow-ups data would have improved our understanding regarding PMT behavior, defining risks and beneficial effects of medical treatment in favor of surgical.

This study has several strengths and limitations. First of all, the prospective registration of the systematic review in PROSPERO enhances methodological transparency of the study. Sequentially, the design of the study combining the clinical survey and the systematic review fit the most the rarity of the condition (2, 3). As a clinical survey of one center experience, it allows to prospectically collect several patients followed for more than 10 years. On the other hand, the systematic review design advantages the study by guaranteeing a collection of large amounts of data, independently extracted by multiple authors with consensus resolution. However, they lack of homogeneity, limiting the clinical values of the same that can only be described. In addition, differently defined outcome measurement and missing data reduced the data collected, and not all the variables couldn’t be properly analyzed, mostly referring to post-treatment outcomes.

Lastly, an important consideration concerns the potential risk of bias within the study, which may have influenced the magnitude of the observed effects. In particular, missing data and their heterogeneity across sources limit the robustness and interpretability of the findings. Missing or inconsistently reported data, and the predominantly observational nature of the evidence may have introduced uncertainty in the estimation of the effect size, potentially leading to either an overestimation or underestimation. In addition, the lack of a stratified analysis for sensitivity by study design, prevents an assessment of the robustness of the findings. As result, caution should be warranted in the interpretation of the findings. Further well-designed studies with more comprehensive data reporting and appropriate robustness analyses are needed to confirm and clarify the observed associations.

As described in our clinical survey and systematic review, burosumab represents a safe and effective treatment for TIO patients who have no surgical treatment option or who are unresponsive to conventional therapy. Given its safety, it may be administered both in cases of tumor identification while waiting for surgery, both in case of surgical inoperability to manage hormonal FGF23 effect and PMTs evolution. Further studies are need to clarify if the use of burosumab may reduce the malignant evolution of PMTs (3).

## Data Availability

The raw data supporting the conclusions of this article will be made available by the authors, without undue reservation.
