# Supplementary material for: Burosumab an emerging therapy in TIO: a local clinical survey and a systematic review with individual patient data analysis
Source: Front Endocrinol (Lausanne). 2026 Apr 2;17:1804013. doi: 10.3389/fendo.2026.1804013 (PMC13082987; doi:10.3389/fendo.2026.1804013)
Supplement: Supplementary file 1 [file Table1.docx]

Supplementary Material

**Supplementary Materials: Table 1. Quality assessment for case reports.**

|  | **JBI Critical Appraisal Checklist for Case Reports** | | | | | | | |
| --- | --- | --- | --- | --- | --- | --- | --- | --- |
| **Ref.** | **1** | **2** | **3** | **4** | **5** | **6** | **7** | **8** |
|  | Y | Y | Y | Y | Y | Y | Y | Y |
|  | Y | Y | Y | Y | Y | Y | Y | Y |
|  | N | N | N | N | N | Y | N | Y |
|  | Y | Y | Y | Y | Y | Y | Y | Y |
|  | Y | Y | Y | Y | Y | Y | Y | Y |
|  | Y | Y | Y | Y | Y | Y | Y | Y |
|  | Y | Y | Y | Y | Y | Y | Y | Y |
|  | Y | Y | Y | Y | Y | Y | Y | Y |
|  | Y | Y | Y | Y | Y | Y | Y | Y |
|  | Y | Y | N | Y | Y | N | N | Y |
|  | Y | Y | Y | Y | Y | Y | Y | Y |
|  | Y | Y | Y | Y | Y | Y | Y | Y |
|  | Y | Y | Y | Y | Y | Y | Y | Y |
|  | Y | N | Y | N | Y | Y | Y | Y |
|  | N | N | Y | N | N | N | Y | Y |
|  | N | N | Y | N | N | N | N | Y |
|  | N | N | Y | N | N | N | N | Y |
|  | Y | Y | Y | Y | Y | Y | Y | Y |
|  | N | N | Y | N | N | N | N | Y |
|  | Y | Y | Y | Y | N | N | N | Y |
|  | Y | Y | Y | N | N | N | N | Y |
|  | Y | Y | Y | Y | Y | Y | Y | Y |
|  | Y | Y | Y | N | Y | Y | Y | Y |
|  | Y | Y | Y | Y | Y | Y | Y | Y |
|  | Y | Y | Y | Y | Y | Y | Y | Y |
|  | Y | Y | Y | Y | Y | Y | Y | Y |
|  | Y | Y | Y | Y | N | N | N | Y |
|  | Y | N | Y | N | Y | Y | Y | Y |
|  | Y | Y | Y | Y | N | N | Y | Y |
|  | Y | Y | Y | Y | Y | Y | Y | Y |
|  | Y | Y | Y | Y | Y | Y | Y | Y |
|  | Y | Y | Y | Y | Y | Y | Y | Y |
|  | Y | Y | Y | Y | Y | Y | Y | Y |
|  | Y | Y | Y | Y | Y | Y | Y | Y |
|  | Y | Y | Y | Y | Y | Y | Y | Y |
|  | Y | Y | Y | Y | Y | Y | Y | Y |
|  | Y | Y | Y | Y | Y | Y | Y | Y |

1. Were patient’s demographic characteristics clearly described?
2. Was the patient’s history clearly described and presented as a timeline?
3. Was the current clinical condition of the patient on presentation clearly described?
4. Were diagnostic tests or assessment methods and the results clearly described?
5. Was the intervention(s) or treatment procedure(s) clearly described?
6. Was the post-intervention clinical condition clearly described?
7. Were adverse events (harms) or unanticipated events identified and described?
8. Does the case report provide takeaway lessons?

Ref: references. Yes: Y. No: N. U: Unclear. Not applicable: N.A.

Moola S, Munn Z, Tufanaru C, Aromataris E, Sears K, Sfetcu R, Currie M, Lisy K, QureYshi R, Mattis P, Mu P. Chapter 7: Systematic reviews of etiology and risk. In: Aromataris E, Munn Z (Editors)*. JBI Manual for Evidence Synthesis.* JBI, 2020. Available from https://synthesismanual.jbi.global. <https://doi.org/10.46658/JBIMES-20-08>

**References**

1. Abebe L, Phung K, Robinson ME, Waldner R, Carsen S, Smit K, Tice A, Lazier J, Armour C, Page M, Dover S, Rauch F, Koujok K, Ward LM. Burosumab for the treatment of cutaneous-skeletal hypophosphatemia syndrome. Bone Rep. 2023 Nov 11;20:101725. doi: 10.1016/j.bonr.2023.101725. PMID: 38229908; PMCID: PMC10790024.
2. Barai R, Tsang T, Cespedes L. Tumour-induced osteomalacia due to residual benign glomangioma. BMJ Case Rep. 2022 Nov 10;15(11):e250237. doi: 10.1136/bcr-2022-250237. PMID: 36357106; PMCID: PMC9660516.
3. Cadiou S, Chapurlat R, Couture G, Guggenbuhl P, Guillot P, Javier RM, Mehsen N, Morizot C, Trijau S, Paccou J. Real-world efficacy and safety of burosumab in tumor-induced osteomalacia: case series from an early access program. JBMR Plus. 2025 Mar 10;9(6):ziaf039. doi: 10.1093/jbmrpl/ziaf039. PMID: 40329993; PMCID: PMC12050030.
4. Crotti C, Zucchi F, Alfieri C, Caporali R, Varenna M. Long-term use of burosumab for the treatment of tumor-induced osteomalacia. Osteoporos Int. 2023 Jan;34(1):201-206. doi: 10.1007/s00198-022-06516-6. Epub 2022 Aug 4. PMID: 35925260; PMCID: PMC9361946.
5. Day AL, Gutiérrez OM, Guthrie BL, Saag KG. Burosumab in tumor-induced osteomalacia: A case report. Joint Bone Spine. 2020 Jan;87(1):81-83. doi: 10.1016/j.jbspin.2019.07.012. Epub 2019 Aug 3. PMID: 31382017.
6. Horinouchi Y, Shiota S, Kaimori R, Yoshimura K, Utsunomiya-Nishimizu R, Yamamoto K, Miyazaki E. A Case of Tumor-Induced Osteomalacia Detected by Venous Sampling. Int Med Case Rep J. 2023 Oct 10;16:659-665. doi: 10.2147/IMCRJ.S425599. PMID: 37840970; PMCID: PMC10576504.
7. Ito N, Fukumoto S. Tumor-induced rickets/osteomalacia (TIO): diagnostic pitfalls and therapeutic options. J Bone Miner Res. 2025 May 24;40(5):572-576. doi: 10.1093/jbmr/zjaf047. PMID: 40156290.
8. Lasnier-Siron J, Perret R, Jaafar A, Schaeverbeke T, Cetre NM. Oncogenic rickets diagnosed at age 8 and the risk of persistent rickets: a rare case of pediatric-onset tumor-induced osteomalacia. Osteoporos Int. 2025 Aug;36(8):1481-1485. doi: 10.1007/s00198-025-07469-2. Epub 2025 May 13. PMID: 40358681; PMCID: PMC12373540.
9. Miyaoka D, Imanishi Y, Yano M, Toi N, Nagata Y, Kurajoh M, Yamada S, Morioka T, Emoto M. Effects of burosumab on osteocalcin and bone mineral density in patient with 15-year history of nonremission tumor-induced osteomalacia initially treated with conventional therapy: Case report. Bone Rep. 2020 Nov 21;13:100736. doi: 10.1016/j.bonr.2020.100736. PMID: 33294501; PMCID: PMC7701316.
10. Nguyen MK, Bandaru D, Nguyen MK. Hyperparathyroidism Secondary to Burosumab Treatment. Cureus. 2025 Aug 7;17(8):e89569. doi: 10.7759/cureus.89569. PMID: 40922882; PMCID: PMC12413978.
11. Oe Y, Kameda H, Nomoto H, Sakamoto K, Soyama T, Cho KY, Nakamura A, Iwasaki K, Abo D, Kudo K, Miyoshi H, Atsumi T. Favorable effects of burosumab on tumor-induced osteomalacia caused by an undetectable tumor: A case report. Medicine (Baltimore). 2021 Nov 19;100(46):e27895. doi: 10.1097/MD.0000000000027895. PMID: 34797338; PMCID: PMC8601343.
12. Paz-Ibarra J, Sáenz-Bustamante S, Inostroza-Fernández M, Hermenegildo PS, Lescano LA, Concepción-Zavaleta M, Román-González A, Reza-Albarrán AA. Acquired hypophosphatemic osteomalacia: case series from a Peruvian referral center (1999-2023). Arch Osteoporos. 2024 Nov 19;19(1):116. doi: 10.1007/s11657-024-01476-2. Erratum in: Arch Osteoporos. 2024 Dec 6;20(1):1. doi: 10.1007/s11657-024-01481-5. Erratum in: Arch Osteoporos. 2025 Jul 15;20(1):92. doi: 10.1007/s11657-025-01583-8. PMID: 39560849.
13. Ramzan A, Lodhi FAK, Ramzan F. Refractory Hypophosphatemia Secondary to Tumor-Induced Osteomalacia: Diagnostic Challenges and Successful Management With Burosumab. Cureus. 2025 Aug 13;17(8):e90016. doi: 10.7759/cureus.90016. PMID: 40951211; PMCID: PMC12431869.
14. Song C, Chan D, Flaman A, Kurien E, Kline G, Ghaznavi S. Burosumab treatment of tumour-induced osteomalacia from a rib lesion. BMJ Case Rep. 2025 May 15;18(5):e265058. doi: 10.1136/bcr-2025-265058. PMID: 40379296.
15. Jan de Beur SM, Cimms T, Nixon A, Theodore-Oklota C, Luca D, Roberts MS, Egan S, Graham CA, Hribal E, Evans CJ, Wood S, Williams A. Burosumab Improves Patient-Reported Outcomes in Adults With Tumor-Induced Osteomalacia: Mixed-Methods Analysis. J Bone Miner Res. 2023 Nov;38(11):1654-1664. doi: 10.1002/jbmr.4900. Epub 2023 Sep 4. PMID: 37578099.
16. Hidaka N, Koga M, Kimura S, Hoshino Y, Kato H, Kinoshita Y, Makita N, Nangaku M, Horiguchi K, Furukawa Y, Ohnaka K, Inagaki K, Nakagawa A, Suzuki A, Takeuchi Y, Fukumoto S, Nakatani F, Ito N. Clinical Challenges in Diagnosis, Tumor Localization and Treatment of Tumor-Induced Osteomalacia: Outcome of a Retrospective Surveillance. J Bone Miner Res. 2022 Aug;37(8):1479-1488. doi: 10.1002/jbmr.4620. Epub 2022 Jul 1. PMID: 35690913.
17. Thomas O Carpenter, Paul D Miller, Thomas J Weber, Munro Peacock, Karl L Insogna, Rajiv Kumar, Diana Luca, Tricia Cimms, Mary Scott Roberts, Suzanne Jan de Beur, OR29-06 Burosumab Improves Biochemical, Skeletal, and Clinical Features of Tumor-Induced Osteomalacia Syndrome, *Journal of the Endocrine Society*, Volume 4, Issue Supplement_1, April-May 2020, OR29–06, <https://doi.org/10.1210/jendso/bvaa046.403>
18. Aliberti L, Gagliardi I, Pontrelli M, Zatelli MC, Ambrosio MR. Tumour-induced osteomalacia: 18 months of 2-weekly burosumab treatment. Endocrinol Diabetes Metab Case Rep. 2023 Jan 25;2023(1):22-0317. doi: 10.1530/EDM-22-0317. PMID: 37931408; PMCID: PMC9986401.
19. Crotti C, Bartoli F, Coletto LA, Manara M, Marini E, Daolio PA, Parafioriti A, Armiraglio E, Zucchi F, Sinigaglia L, Caporali R, Varenna M. Tumor induced osteomalacia: A single center experience on 17 patients. Bone. 2021 Nov;152:116077. doi: 10.1016/j.bone.2021.116077. Epub 2021 Jun 25. PMID: 34175499.
20. Burosumab Effects on Osteomalacia: A Tumor-Induced Osteomalacia (TIO) Case Report PUB078. Foligno, Nadia Edvige; Kato, Hajime; Hoshino, Yoshitomo; Kimura, Soichiro; Hidaka, Naoko; Sunouchi, Takashi; Watanabe, So; Arcidiacono, Teresa; Bologna, Arianna; Giambò, Federica; Vezzoli, Giuseppe; Ito, Nobuaki. Journal of the American Society of Nephrology 34(11S):p 1070-1071, November 2023. | DOI: 10.1681/ASN.20233411S11070d
21. Yi Shan Der, Santosh Kumar Chaubey, Ashim Kumar Sinha, 6542 Burosumab In Unresectable/Unidentifiable Tumor-Induced Osteomalacia, *Journal of the Endocrine Society*, Volume 8, Issue Supplement_1, October-November 2024, bvae163.375, <https://doi.org/10.1210/jendso/bvae163.375>
22. Toi N, Imanishi Y, Nagata Y, Kurajoh M, Morioka T, Shoji T, Shinto Y, Emoto M. Improvement in the mobility of a patient with fibroblast growth factor 23-related hypophosphatemic osteomalacia and decompensated liver cirrhosis in response to burosumab: a case report. Endocr J. 2023 Apr 28;70(4):419-426. doi: 10.1507/endocrj.EJ22-0520. Epub 2022 Dec 28. PMID: 36575021.
23. Yoshida S, Yatsuzuka K, Fujibuchi T, Nakaguchi H, Kohri N, Muto J, Shiraishi K, Murakami M and Fujisawa Y (2024) Adult epidermal nevus syndrome with hypophosphatemic osteomalacia treated with burosumab: a case study and literature review. J. Cutan. Immunol. Allergy 7:12575. doi: 10.3389/jcia.2024.12575
24. Tumour induced osteomalacia: 2 years treatment with burosumab. Ludovica Aliberti , Margherita Pontrelli , Martina Verrienti , Irene Gagliardi , Maria Chiara Zatelli & Maria Rosaria Ambrosio
25. Koichi Okajima, Hiroshi Kobayashi, Hayata Suzuki, Liuzhe Zhang, Yuki Ishibashi, Yusuke Tsuda, Munetoshi Hinata, Yoichi Yasunaga, Tetsuo Ushiku, Yuji Nakamoto, Yasuhiro Takeuchi, Sakae Tanaka, Progression of phosphaturic mesenchymal tumor in the femoral bone during treatment with burosumab: A case report, JOS Case Reports, Volume 3, Issue 1, 2024, Pages 6-9, ISSN 2772-9648, <https://doi.org/10.1016/j.joscr.2023.09.011>.
26. Tratamiento con burosumab en osteomalacia oncogénica: a propósito de un caso. March 2023. [Fronteras en Medicina](https://www.researchgate.net/journal/Fronteras-en-Medicina-2618-2521?_tp=eyJjb250ZXh0Ijp7ImZpcnN0UGFnZSI6InB1YmxpY2F0aW9uIiwicGFnZSI6InB1YmxpY2F0aW9uIn19) 18(01) DOI:[10.31954/RFEM/202301/0042-0047](https://doi.org/10.31954/RFEM/202301/0042-0047)
27. Chiu HS, Tsai MM, Wang TM, Lee NC, Tung YC. Effective bone healing after corrective osteotomy in a patient with FGF23-related hypophosphatemic disease using short-term burosumab treatment. J Formos Med Assoc. 2025 Jan;124(1):87-90. doi: 10.1016/j.jfma.2024.10.004. Epub 2024 Oct 6. PMID: 39375070.
28. A LARGE AGGRESSIVE PHOSPHATURIC MESENCHYMAL TUMOR OF THE HUMERUS
29. SURGICAL MANAGEMENT AND BUROSUMAB THERAPY FOR TUMOR-INDUCED OSTEOMALACIA.Tasma Harindhanavudh Andrea Espejo-Freire Paari Murugan Edward Cheng DOI:<https://doi.org/10.15605/jafes.038.AFES.59>
30. Pinto Valadares
31. Yamamoto K, Honda H, Ota I, Otsuka F. Triad signs shown by bone scintigraphy in FGF23-related osteomalacia. QJM. 2022 Jan 9;114(12):887-888. doi: 10.1093/qjmed/hcab240. PMID: 34554259.
32. Charlotte R Lichtenfeld, Lucase Rehm, Luke T Drake, Scout S Dahir, Kathryn McCrystal Dahir, SUN-755 Tumor Induced Osteomalacia Caused by Metastatic Thyroid Cancer, *Journal of the Endocrine Society*, Volume 9, Issue Supplement_1, October-November 2025, bvaf149.705, <https://doi.org/10.1210/jendso/bvaf149.705>
33. Merz LM, Buerger F, Ziegelasch N, Zenker M, Wieland I, Lipek T, Wallborn T, Terliesner N, Prenzel F, Siekmeyer M, Dittrich K. A Case Report: First Long-Term Treatment With Burosumab in a Patient With Cutaneous-Skeletal Hypophosphatemia Syndrome. Front Endocrinol (Lausanne). 2022 May 6;13:866831. doi: 10.3389/fendo.2022.866831. PMID: 35600592; PMCID: PMC9120998.
34. Khadora M, Mughal MZ. Burosumab treatment in a child with cutaneous skeletal hypophosphatemia syndrome: A case report. Bone Rep. 2021 Oct 1;15:101138. doi: 10.1016/j.bonr.2021.101138. PMID: 34660853; PMCID: PMC8502709.
35. Ghanta
36. A Rare Case of Tumor-Induced Osteomalacia Despite Resection of a Benign Glomangioma Author links open overlay panel Tiffany Tsang, Lissette Cespedes MD
37. Del Pino M, Viterbo G, Valentini MA, Salvatierra R, Belleri F, La Forgia P, Abbate S, Chinton J, Lazzati JM, Maderna O, Cervini AB, Fano V. Burosumab Treatment in a Girl With Cutaneous Skeletal Hypophosphatemia Syndrome: 2-Year Follow-Up. Am J Med Genet A. 2025 Jun;197(6):e64020. doi: 10.1002/ajmg.a.64020. Epub 2025 Feb 10. PMID: 39925163.

**Supplementary Materials: Table 2. Quality assessment for case series.**

|  | **JBI Critical Appraisal Checklist for Case Series** | | | | | | | | | |
| --- | --- | --- | --- | --- | --- | --- | --- | --- | --- | --- |
| Ref. | 1 | 2 | 3 | 4 | 5 | 6 | 7 | 8 | 9 | 10 |
|  | Y | Y | Y | N | Y | Y | Y | N | N | NA |
|  | Y | Y | Y | Y | Y | Y | Y | Y | Y | NA |
|  | Y | N | Y | N | N | N | N | Y | Y | NA |
|  | Y | Y | Y | Y | Y | Y | Y | Y | Y | Y |

1. Were there clear criteria for inclusion in the case series?
2. Was the condition measured in a standard, reliable way for all participants included in the case series?
3. Were valid methods used for identification of the condition for all participants included in the case series?
4. Did the case series have consecutive inclusion of participants?
5. Did the case series have complete inclusion of participants?
6. Was there clear reporting of the demographics of the participants in the study?
7. Was there clear reporting of clinical information of the participants?
8. Were the outcomes or follow-up results of cases clearly reported?
9. Was there clear reporting of the presenting sites’/clinics’ demographic information?
10. Was statistical analysis appropriate?

Yes: Y. No: N. Unclear: U. Not Applicable: N.A.

Moola S, Munn Z, Tufanaru C, Aromataris E, Sears K, Sfetcu R, Currie M, Lisy K, Qureshi R, Mattis P, Mu P. Chapter 7: Systematic reviews of etiology and risk. In: Aromataris E, Munn Z (Editors)*. JBI Manual for Evidence Synthesis.* JBI, 2020. Available from https://synthesismanual.jbi.global. https://doi.org/10.46658/JBIMES-20-08

**Reference**

1. Nagata D, Takashi Y, Yamamoto M, Toyokawa K, Makihata K, Koganemaru H, Hideshima S, Muta Y, Yokomizo H, Fukumoto H, Nakayama S, Muta H, Aoki M, Abe H, Yamamoto T, Hamasaki M, Kawanami D. Two Cases of Tumor-induced Osteomalacia Resulting in Surgical Resection during Burosumab Therapy. Intern Med. 2025 Sep 1;64(17):2597-2603. doi: 10.2169/internalmedicine.4958-24. Epub 2025 Mar 8. PMID: 40058863; PMCID: PMC12463426.
2. Sugarman J, Maruri A, Hamilton DJ, Tabatabai L, Luca D, Cimms T, Krolczyk S, Roberts MS, Carpenter TO. The efficacy and safety of burosumab in two patients with cutaneous skeletal hypophosphatemia syndrome. Bone. 2023 Jan;166:116598. doi: 10.1016/j.bone.2022.116598. Epub 2022 Oct 27. PMID: 36341949.
3. Gronskaia SA, Belaya ZE, Rozhinskaya LY, Melnichenko GA, Dubovitskaya TA, Mamedova EO, Rodionova SS, Buklemishev YV, Pigarova EA, Degtyarev MV, Babaeva DM, Vladimirova VP, Tarbaeva NV, Serzhenko SS, Grigoriev AY, Dzeranova LK, Karpenko VY, Karasev AL, Fedotov RN, Uliyanova IN, Toroptsova NV, Lesnyak OM, Mokrysheva NG, Dedov II. [Clinical features, diagnostics and treatment of FGF23 secreting tumors: series of 40 clinical cases]. Probl Endokrinol (Mosk). 2023 Nov 10;69(5):25-38. Russian. doi: 10.14341/probl13221. PMID: 37968949; PMCID: PMC10680540.
4. Cianferotti L, Delli Poggi C, Bertoldo F, Caffarelli C, Crotti C, Gatti D, Giannini S, Gonnelli S, Mazzantini M, Ombretta V, Sella S, Setti A, Varenna M, Zucchi F, Brandi ML. Persistence and recurrence in tumor-induced osteomalacia: A systematic review of the literature and results from a national survey/case series. Endocrine. 2022 Jun;76(3):709-721. doi: 10.1007/s12020-022-03039-2. Epub 2022 Apr 5. PMID: 35381903; PMCID: PMC9156492.

**Supplementary Materials: Table 3. Quality assessment for Clinical Trial (ROBINS-I)**

| Bias due to confounding | Is there potential for confounding of the effect of intervention in this study? | Y | Serious risk of bias |
| --- | --- | --- | --- |
|  | Was the analysis based on splitting participants’ follow up time according to intervention received? | N/NA |  |
|  | Appropriate analysis method controlling for all important confounding domains? | N |  |
|  | Control for post-intervention variables affected by the intervention? |  |  |
| Bias in selection of participants into the study | Selection into the study/analysis based on post-intervention characteristics? | N/PN | Moderate risk of bias |
|  | Do start of follow-up and start of intervention coincide for most participants? | Y/PY |  |
| Bias in classification of interventions | Were intervention groups clearly defined? | Y | Low risk of bias |
|  | Information recorded at start of intervention? | Y/PY |  |
|  | Could classification have been affected by knowledge of outcome? | N/PN |  |
| Bias due to deviations from intended interventions | Important co-interventions balanced across groups? | NI/NA | Moderate risk of bias |
|  | Was the intervention implemented successfully for most participants? | Y/PY |  |
|  | Did participants adhere to the assigned regimen? | Y/PY |  |
| Bias due to missing data | Outcome data available for all, or nearly all, participants? | PN | Moderate risk of bias |
|  | Excluded due to missing intervention status? | N |  |
|  | Excluded due to missing data on other variables needed for the analysis? | N/NI |  |
|  | Robustness to missing data? | N/NI |  |
| Bias in measurement of outcomes | Could outcome be influenced by knowledge of intervention? | N/NP | Low risk of bias |
|  | Were outcome assessors aware? | PY |  |
|  | Comparable assessment methods across groups? | NA |  |
|  | Systematic errors related to intervention? | PN |  |
| Bias in selection of the reported result | Multiple outcome measurements within the domain? | PY/NI | Moderate risk of bias |
|  | Multiple analyses of the intervention–outcome relationship? | PN |  |
|  | Different subgroups? | N/NI |  |
|  | Overall risk of bias: SERIOUS | |  |

Y: yes; PY: probably yes; PN: probably no; N: no; NI: no information; NA: not applicable.

**Reference:**

Imanishi Y, Ito N, Rhee Y, Takeuchi Y, Shin CS, Takahashi Y, Onuma H, Kojima M, Kanematsu M, Kanda H, Seino Y, Fukumoto S. Interim Analysis of a Phase 2 Open-Label Trial Assessing Burosumab Efficacy and Safety in Patients With Tumor-Induced Osteomalacia. J Bone Miner Res. 2021 Feb;36(2):262-270. doi: 10.1002/jbmr.4184. Epub 2020 Nov 4. PMID: 32967046; PMCID: PMC7988547.
